# Increasing Use of Postpartum Family Planning and the Postpartum IUD: Early Experiences in West and Central Africa

**DOI:** 10.9745/GHSP-D-16-00039

**Published:** 2016-08-11

**Authors:** Tsigue Pleah, Yolande Hyjazi, Suzanne Austin, Abdoulaye Diallo, Blami Dao, Rachel Waxman, Priya Karna

**Affiliations:** a Jhpiego, Baltimore, MD, USA; b Jhpiego, Republic of Guinea, Conakry, Guinea; c Johns Hopkins Bloomberg School of Public Health, Baltimore, MD, USA

## Abstract

A global resurgence of interest in the intrauterine device (IUD) as an effective long-acting reversible contraceptive and in improving access to a wide range of contraceptive methods, as well as an emphasis on encouraging women to give birth in health care facilities, has led programs to introduce postpartum IUD (PPIUD) services into postpartum family planning (PPFP) programs. We describe strategic, organizational, and technical elements that contributed to early successes of a regional initiative in West and Central Africa to train antenatal, maternity, and postnatal care providers in PPFP counseling for the full range of available methods and in PPIUD service delivery. In November 2013, the initiative provided competency-based training in Guinea for providers from the main public teaching hospital in 5 selected countries (Benin, Chad, Côte d’Ivoire, Niger, and Senegal) with no prior PPFP counseling or PPIUD capacity. The training was followed by a transfer-of-learning visit and monitoring to support the trained providers. One additional country, Togo, replicated the initiative’s model in 2014. Although nascent, this initiative has introduced high-quality PPFP and PPIUD services to the region, where less than 1% of married women of reproductive age use the IUD. In total, 21 providers were trained in PPFP counseling, 18 of whom were also trained in PPIUD insertion. From 2014 to 2015, more than 15,000 women were counseled about PPFP, and 2,269 women chose and received the PPIUD in Benin, Côte d’Ivoire, Niger, Senegal, and Togo. (Introduction of PPIUD services in Chad has been delayed.) South–South collaboration has been central to the initiative’s accomplishments: Guinea’s clinical centers of excellence and qualified trainers provided a culturally resonant example of a PPFP/PPIUD program, and trainings are creating a network of regional trainers to facilitate expansion. Two of the selected countries (Benin and Niger) have expanded their PPFP/PPUID training programs to additional sites. Inspired after learning about the initiative at a regional meeting, Togo has outperformed the original countries involved in the initiative by training more providers than the other countries. Challenges to scale-up include a lack of formal channels for reporting PPFP and PPIUD service delivery outcomes, inconsistent coordination of services across the reproductive health continuum of care, and slow uptake in some countries. Continued success will rely on careful recordkeeping, regular monitoring and feedback, and strategic data use to advocate scale-up.

## Abstract (precis)

Competency-based training in postpartum family planning and postpartum IUD (PPIUD) service delivery of antenatal, maternity, and postnatal care providers from 5 francophone African countries generated an enthusiastic response from the providers and led to government and donor support for expansion of the approach. More than 2,000 women chose and received the PPIUD between 2014 and 2015. This model of South–South cooperation, when coupled with demand promotion, supportive supervision, and reliable collection of service outcome data, can help to expand PPIUD services in other regions as well.

## BACKGROUND

In low-income countries, increasing emphasis on antenatal care (ANC) and childbirth in a health care facility (institutional delivery) has created an opportunity to counsel women about family planning. The health benefits of contraception and birth spacing for women and their infants are striking.^1^^,^^2^ During the postpartum period, many women want to delay or avoid a subsequent pregnancy, but unmet need for family planning is higher during the first year after childbirth than at any other time.^3^ At 6 weeks postpartum, Pasha et al. found that 95% of women in 5 low-income countries wished to avoid pregnancy for at least 1 year.^4^ Women who attend ANC are more likely to initiate postpartum family planning (PPFP) than those who do not,^5^^,^^6^ which indicates that women are particularly receptive to information about contraception and birth spacing during pregnancy.

Renewed interest in the intrauterine device (IUD), a highly effective, long-acting reversible contraceptive (LARC) that is safe for lactating women, has encouraged some programs to add postpartum IUD (PPIUD) services to their PPFP options.^1^^,^^7^^-^^9^ A PPIUD can be inserted within minutes after delivery of the placenta (postplacental insertion), up to 48 hours after childbirth (immediate postpartum insertion), or during a cesarean delivery (intracesarean insertion). PPIUD insertion may avoid discomfort associated with interval insertion (insertion 4 weeks or more after delivery), and bleeding from insertion will be disguised by postpartum lochia (the normal discharge from the uterus after childbirth).^10^ Historically, postplacental and immediate postpartum insertion have been associated with higher rates of expulsion than interval insertion, but improved insertion techniques have reduced this risk.^1^^,^^10^^,^^11^ IUDs are cost-effective, can be inserted in a matter of minutes by a trained provider, and do not require an additional facility visit when inserted during the childbirth stay.^10^^,^^12^ Insertion before discharge from the birthing facility ensures that the woman is not pregnant at the time of insertion and is protected against pregnancy prior to resuming sexual activity.^1^

Successful PPFP/PPIUD interventions have focused capacity-building efforts on providers of ANC and labor and delivery at high-volume maternity units with a strong record of infection prevention and counseling.

PPIUDs make up a small share of the method mix in sub-Saharan Africa,^13^ but West and Central African countries are investing in efforts to broaden the contraceptive method mix and increase low contraceptive prevalence rates.^14^ Successful PPFP and PPIUD interventions have focused capacity-building efforts on providers of ANC and labor and delivery care at high-volume maternity units with a strong record of infection prevention and counseling.^8^ Evidence from India, Kenya, and Zambia shows that nonphysicians perform PPIUD insertions as well as physicians, with similar outcomes, as long as they receive appropriate competency-based training.^7^^,^^15^ Lessons learned from other PPIUD interventions indicate that service strengthening is crucial, including advocacy, training, provision of key supplies and equipment, demand creation, supportive supervision, and strong monitoring and evaluation (M&E).^8^

In this article, we describe the process of developing and implementing a regional initiative using competency-based training to introduce PPFP and PPIUD services in selected public teaching hospitals in francophone West and Central Africa. We highlight early lessons learned in this nascent initiative that have led to promising uptake and expansion of PPIUD services, while also documenting program and recordkeeping challenges.

## PROGRAM DESCRIPTION

### Preparing the Soil: The Power of Meetings to Spark Interest in PPFP and the PPIUD

In July 2012, donors, governments, and development assistance agencies committed to revitalize investment in family planning services at the London Summit on Family Planning. Countries committed to specific actions, objectives, policy changes, and investments in family planning as part of the Family Planning 2020 (FP2020) initiative spawned by this conference.^14^

A few months later in 2012, technical experts highlighted practical, effective reproductive health interventions, including PPFP and PPIUD services, at a session of the annual congress of the Société Africaine des Gynécologues et Obstétriciens (SAGO) in Niger, and Jhpiego, an international health NGO headquartered in the United States, gave a presentation detailing the benefits of its PPFP/PPIUD implementation strategy and results from successful programs. The presentations sparked a groundswell of enthusiasm from attendees for a PPIUD initiative, including representatives from the United Nations Population Fund (UNFPA) and ministries of health (MOHs) in West and Central African countries.

Drawing upon its program experience in the West and Central African region, Jhpiego subsequently submitted a concept note to the regional UNFPA technical director proposing an initiative to facilitate scale-up of PPFP/PPIUD services to integrate family planning and maternal health services at the facility level. Interested parties continued to consolidate support for a regional PPFP/PPIUD initiative, including giving a presentation at the annual meeting of the Association Sénégalaise de Gynécologie Obstétrique (ASGO) in July 2013.

### Planting the Seed: Designing the PPFP/PPIUD Initiative

**FIGURE 1.**
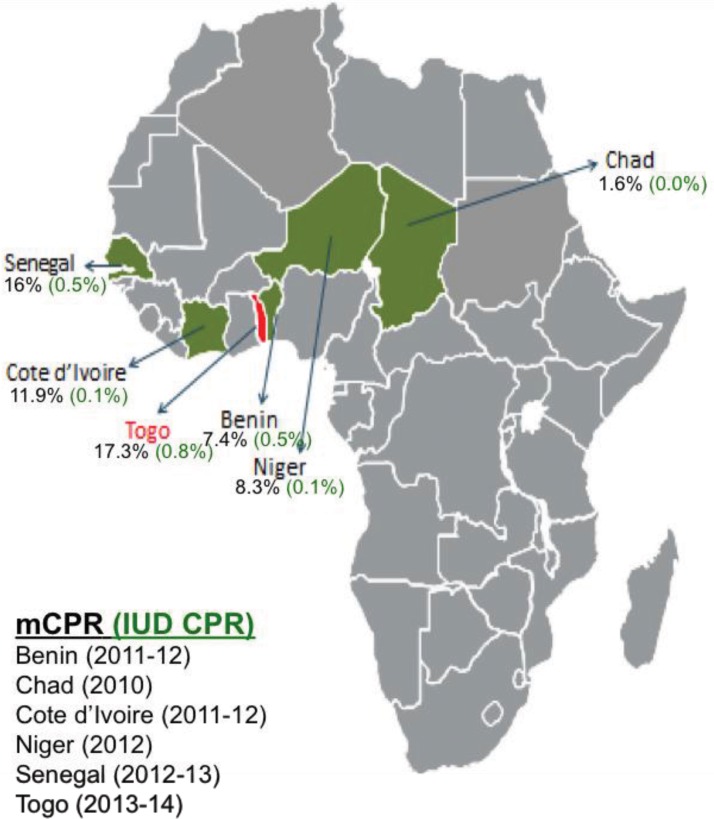
mCPR and IUD CPR in the PPFP/PPIUD Initiative Countries and Togo^a^ Abbreviations: CPR, contraceptive prevalence rate; IUD, intrauterine device; mCPR, modern contraceptive prevalence rate; PPFP, postpartum family planning; PPIUD, postpartum intrauterine device. ^a^ The initiative was launched in 5 countries (Benin, Chad, Côte d’Ivoire, Niger, Senegal) in November 2013; Togo replicated the initiative’s model in 2014. Source: United Nations, Department of Economic and Social Affairs, Population Division.^16^

The goal of the PPFP/PPIUD initiative, which trained providers in November 2013 who then began offering services at the beginning of 2014, was to increase contraceptive prevalence in 5 selected countries where UNFPA had a presence by providing PPFP counseling and services during the antenatal, early labor, and postpartum periods for the full range of methods available, and introducing good-quality PPIUD services in selected health facilities to diversify the PPFP method mix. The 5 countries were Benin, Chad, Côte d’Ivoire, Niger, and Senegal. One additional country, Togo, replicated the initiative’s model in 2014 after hearing about it at a regional PPIUD conference in Ouagadougou, Burkina Faso, in February 2014. After learning that UNFPA had funding for PPFP/PPIUD programs in the region, delegates from Togo approached their in-country UNFPA office and procured funds for their own program. Modern contraceptive prevalence in these 6 countries is low, ranging from 1.6% in Chad to 17.3% in Togo, with IUD usage rates almost nonexistent (<1%) (Figure 1).^16^ At baseline, PPFP/PPIUD services were not available in these countries.

In addition to awareness raising, sensitization, and advocacy, the principal components of the PPIUD initiative included:

1. Provider training
2. Supportive supervision for trained staff
3. Ongoing monitoring of outputs and outcomes

The vision was that the countries would assume ownership for implementing the initiative. Programs in each country would generate data to demonstrate the feasibility and acceptability of services and use these data to advocate sustainable expansion, supported by in-country UNFPA offices and other donors.

#### Regional Competency-Based Training of Country Teams

In November 2013, we held a training session to strengthen providers’ family planning counseling skills and equip them to introduce PPFP and PPIUD services. In each of the 5 selected countries involved in the initiative, a team of 5 health care providers, comprising 2 providers in ANC or postnatal care (PNC), 2 in labor and delivery, and 1 physician in charge of the maternity ward, from a single facility—usually a main public teaching hospital—was invited to attend the training session.

**Figure:**
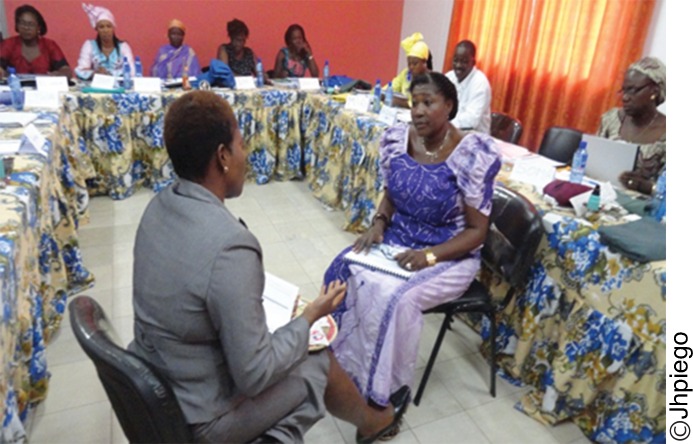
Trainees from West and Central Africa practice postpartum family planning counseling at a training event.

Respected teaching faculty with high caseloads were invited because they had the potential to become competent and influential trainers and ambassadors for the PPIUD (so-called “champion” providers) who could change perceptions and practices in their countries and help expand the initiative.^1^ Guinea was chosen as the training location because Jhpiego had previously supported the implementation of PPFP/PPIUD services in its national hospitals and nearby communal medical centers, which provided an excellent platform for hands-on, experiential training. (The Togo team also requested technical assistance from the PPFP/PPIUD initiative for training of trainers by the Guinea team.)

The training course used a competency-based approach and covered counseling skills, PPIUD insertion techniques, management of side effects and complications, and infection prevention procedures. ANC and PNC providers attended training for counseling only, whereas the labor and delivery providers on each team were trained in PPIUD insertion as well as counseling. Classroom sessions were followed by practice on anatomical models of the postpartum uterus. Anatomical models were furnished for training, and each facility team was given at least 3 instrument kits for PPIUD insertions. The postpartum uterus model differs from a normal uterine anatomical model in how the fundus feels and in the vagino–uterine angle, which must be reduced for proper PPIUD placement. Participants’ performance was assessed using a standardized checklist of skills for successful counseling and insertion.

Four trainers—themselves trained as trainers by Jhpiego—coached participants until they demonstrated competence with the anatomical model, after which the participants visited 1 of 3 practicum sites in Guinea to gain clinical experience performing PPIUD insertions on clients. The participants performed 22 PPIUD insertions: 18 postplacental insertions, 3 intracesarean insertions, and 1 immediate postpartum insertion. The same standardized performance checklist used during training was also used at practicum sites to observe practice and qualify providers on PPIUD insertion with clients.

#### Post-Training Transfer-of-Learning Visit

Following the training, the trainers conducted one formal transfer-of-learning visit at each hospital to monitor the new skills and services and to help providers address challenges in putting their new skills into practice and in managing service delivery. Only one formal visit was possible due to budget constraints. During these visits, they observed providers at work and used standardized checklists to give feedback; examined client flow, organization of services, and the procurement system; and worked to strengthen data collection and reporting. Use of standardized checklists offered an opportunity to improve quality of services. This was also an opportunity to advocate ongoing support for the integration of family planning and maternity services and expansion of the PPFP/PPIUD initiative in each country. Whenever trainers were near one of the hospitals at other points in time, they also visited the providers informally to answer questions, monitor progress, and provide support.

#### Monitoring and Evaluation

The initiative worked with the 5 country teams to develop and strengthen monitoring systems for both PPFP counseling and PPIUD insertions. PPFP and PPIUD indicators are not yet included in any of the countries’ health management and information system (HMIS). Instead, an M&E staff person from Guinea oriented trainees on the use of a standardized logbook provided during the training and sensitized country teams to the importance of accurate data collection and reporting. The site visits by the trainers were also an opportunity to identify gaps in service delivery data collection and errors in recordkeeping, such as discrepancies between the time when a woman was counseled and when she received the PPIUD. However, monitoring efforts suffered from the lack of a dedicated person to collect and collate the data, as well as a lack of accountability inherent in the HMIS. As a result, there are considerable missing data.

## RESULTS

### Growing Buds: Encouraging Signs of Success

The initiative is still in its infancy, and thus it is too early to detect any increase in contraceptive prevalence attributable to the initiative. However, training outputs and service delivery outcomes have been promising, and the program is expanding in the region. During the initial round of training, 21 providers from the 5 countries were instructed in PPFP counseling, 18 of whom were also trained in PPIUD insertion. Although providers from Chad have been trained, introduction of PPIUD services has been delayed, likely due to low ANC coverage, low modern contraceptive prevalence, and a lack of government and agency support. Progress in the other countries has been slow, but the number of sites providing PPFP and PPIUD services has increased from the original 4 in early 2014 to 19 as of January 2016, which includes 7 sites in Togo.

The number of sites providing PPFP and PPIUD services has increased from the original 4 in early 2014 to 19 as of January 2016.

The initiative’s M&E staff person in Guinea collected service data informally from 2014 to 2015, which are presented in the Table for 5 of the 6 countries. Service data for Chad are not provided in the Table due to the delays experienced there. The data point to the following program results:

**TABLE:** PPIUD Service Statistics by Country,^a^ 2014–2015

Service Statistic	Benin		Côte d’Ivoire		Niger		Senegal		Togo		Total		Grand Total
	2014	2015	2014	2015	2014	2015	2014	2015	2014	2015	2014	2015	
Overall													
Cumulative no. of facilities providing PPFP/PPIUD services	1	5	1	1	1	5	1	1	0	7	4	19	19
No. of women receiving PPFP counseling	4,858	2,934	3,829	2,953	NA	NA	NA	820	NA	NA	8,687	6,707	15,394
No. of PPIUD insertions	169	263	199	248	200	247	55	172	125	591	748	1,521	2,269
No. of PPIUD expulsions^b^	1	4	0	4	0	0	0	2	0	8	1	18	19
Timing of counseling for women choosing the PPIUD													
During ANC	20	52	11	59	5	39	0	0	0	0	36	150	186
Before active phase of labor	92	149	61	121	168	97	7	18	0	0	328	385	713
Immediately postpartum	54	62	24	54	25	25	48	154	0	0	151	295	446
Missing data	3	0	103	14	2	86	0	0	125	591	233	691	924
Timing of insertion of the PPIUD													
Postplacental (within 10 minutes after delivery)	29	78	24	50	31	82	1	14	29	124	114	348	462
Immediate postpartum (>10 minutes to 48 hours postpartum)	77	79	26	59	39	13	40	154	30	152	212	457	669
Intracesarean	63	106	47	119	129	152	14	4	4	109	257	490	747
Missing data	0	0	102	20	1	0	0	0	62	206	165	226	391
Method of follow-up consultation among women choosing the PPIUD													
At the facility 4–6 weeks after delivery	28	11	24	115	3	50	30	35	0	16	85	227	312
By telephone 6 weeks or more after delivery	24	45	17	95	134	137	22	37	0	139	197	453	650
No follow-up	117	207	158	38	63	60	3	100	125	436	466	841	1,307

Abbreviations: PPFP, postpartum family planning; PPIUD, postpartum intrauterine device; NA, not available.

a The PPFP/PPIUD initiative was launched in 5 countries—Benin, Chad, Côte d’Ivoire, Niger, and Senegal—with Togo joining later. No data are provided for Chad because introduction of PPIUD services has been delayed there.

b As reported by PPIUD users.

**FIGURE 2.**
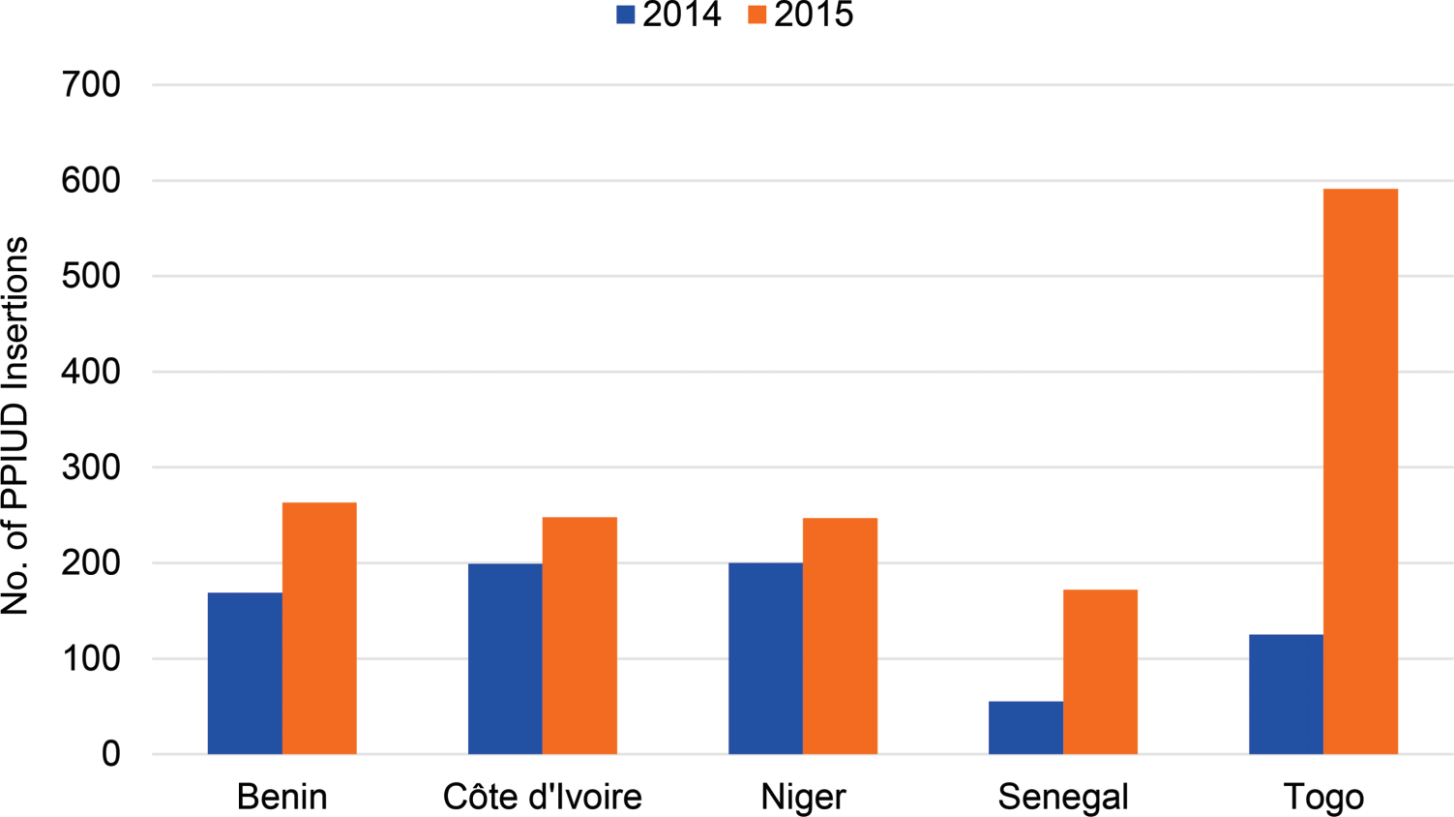
Number of PPIUD Insertions in 5 Countries^a^ in 2014 and 2015 Abbreviations: PPIUD, postpartum intrauterine device. ^a^ Benin, Côte d’Ivoire, Niger, and Senegal were part of the original PPFP/PPIUD initiative; Togo replicated the model afterward. Data for Chad, the fifth initiative country, were not available.

- More than 15,000 women were counseled on PPFP services in Benin, Côte d’Ivoire, Niger, Senegal, and Togo between 2014 and 2015. Year-over-year decreases in numbers of women counseled were observed in some sites, probably due to having met an initial surge in demand after services were first introduced.
- 2,269 of these women in these 5 countries chose to have a PPIUD inserted. The number of women choosing to have a PPIUD inserted increased between 2014 and 2015 in each of the 5 countries with data (Figure 2).
- 0.8% of these 2,269 women reported spontaneous expulsions, which is very low compared with rates of 1.7% to 3.7% reported in similar programs in Ethiopia, Guinea, Pakistan, and the Philippines.^1^
- 42.4% of PPIUD recipients received a follow-up consultation, either in person at 4–6 weeks postpartum (13.8%) or by phone at 6 weeks or after if the woman had not received follow-up and a phone number was on file (28.6%).
- Only 12 women (0.5% of those who had a PPIUD inserted) have requested removal since insertion, 10 of whom expressed a desire to become pregnant and 2 whose husbands disapproved of the IUD (data not shown).

More than 2,000 women in 5 countries chose and had a PPIUD inserted between 2014 and 2015.

**FIGURE 3.**
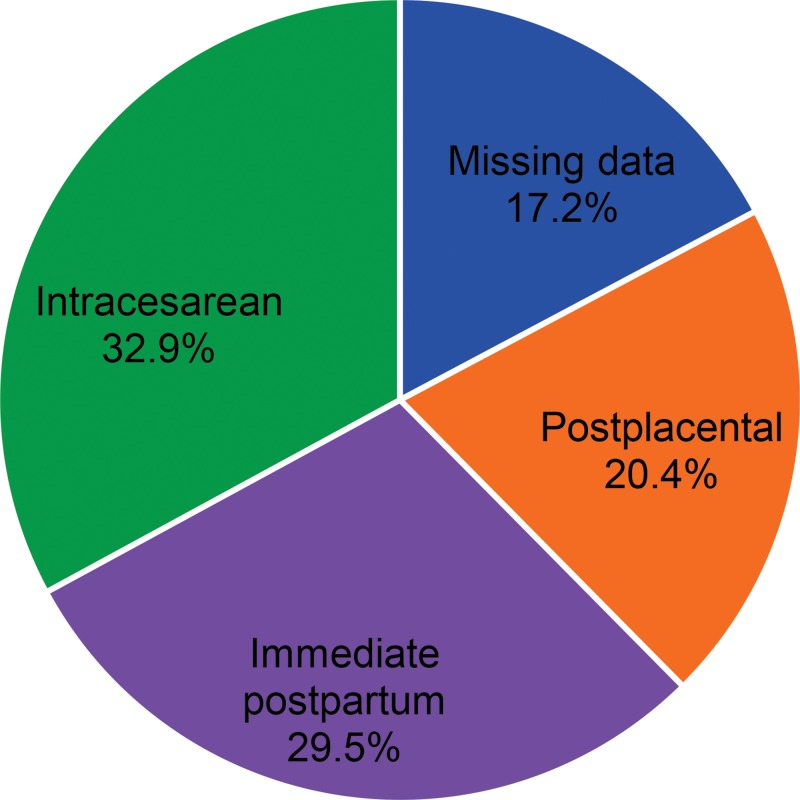
Timing of PPIUD Insertions Among Women Choosing the PPIUD in 5 Countries^a^ Between 2014 and 2015 (N=2,269 Insertions) Abbreviations: PPIUD, postpartum intrauterine device. ^a^ Benin, Côte d’Ivoire, Niger, Senegal, and Togo; data for Chad were not available.

The most common timing for PPIUD insertion to date in the 4 countries with such data is during cesarean delivery (32.9%) (Figure 3). The large proportion of intracesarean insertion is due to 3 countries (Côte d’Ivoire, Benin, and Niger), where physicians who perform cesareans—rather than nonphysician clinicians who cannot—are the most motivated and engaged PPIUD providers. The relatively low percentage of postplacental insertions (20.4%) may be an indicator of weak PPFP counseling at ANC clinics, assuming that the women choosing the PPIUD had made ANC visits. (ANC coverage of at least one visit is high in the West African countries involved in the initiative, ranging from 84% to 87%, and lower in the Central African countries, ranging from 39% to 46%.^17^) If ANC counseling can be strengthened, the share of postplacental insertions should increase.

Only 20% of women choosing the PPIUD had it inserted immediately after birth, potentially suggesting weak PPFP counseling at ANC clinics.

Since launching its program, Togo has outperformed the original countries involved in the initiative, with 13 trainers updated in PPFP and 46 providers trained in PPFP counseling. Of these 46 providers, 33 were trained on PPIUD insertion. Collectively, they have performed 716 PPIUD insertions to date. The adoption by Togo provides a model for country-led introduction and expansion of good-quality PPFP/PPIUD services.

Adoption of the initiative by Togo provides a model for country-led introduction and expansion of PPFP/PPIUD services.

## LESSONS LEARNED

### Meetings Are Opportunities

Experience with developing and rapidly implementing this PPFP/PPIUD initiative across 6 countries in West and Central Africa speaks to the potential of strategic synergies of stakeholders and good ideas at regional and international conferences, as well as the power of data to spur decision makers to action. Critical inputs included:

- Facilitation of the initiative in multiple countries of the region by a respected technical assistance provider
- Cultivation of partnerships between donors, national MOHs, and other key stakeholders
- Sharing of convincing scientific data and program experiences to ignite interest

The effectiveness of meetings to motivate and connect countries with resources to take action is evidenced by Togo’s rapid and successful embrace of the PPFP/PPIUD approach after the 2014 conference in Ouagadougou**.**

### South–South Collaboration Increases Commitment

The prevailing spirit of the initiative is South–South collaboration, increasingly hailed as a successful model for sustainable knowledge and skill transfer.^18^^-^^20^ Training and support from experienced providers in their own region resonates more strongly than training from external providers, particularly as these local experts had themselves been trained by a world-renowned institution. The initial training was based in Guinea because of its experience with PPFP/PPIUD services, its clinical centers of excellence in which to conduct the practicum, and the presence of motivated, culturally similar trainers. Trainees were inspired by observing during their training a successful model for PPFP/PPIUD services in Guinea and also by building rapport with the trainers; they returned home believing that if a PPFP/PPIUD intervention could succeed in such a similar cultural and geographic context, it should be possible in their own country. Shared culture and language also facilitated the training, reduced miscommunication between teams, and built engagement and mutual support. Continued support and guidance from these Guinea-based trainers in informal follow-up visits and phone calls has maintained this enthusiasm and confidence.

Developing a pool of regional trainers is a future goal of the initiative to maximize the potential for future South–South collaboration and further expansion in the region.

The launch of a new country initiative in Togo is an additional example of South–South collaboration. Armed with information, financial resources from UNFPA, and technical support from the Guinea-based trainers, Togo was able to mobilize resources to meet the needs of its people. Developing a pool of regional trainers—a future goal of the initiative—maximizes the potential for future South–South collaboration and builds a platform for further expansion of the program in the region. There are now 15 PPIUD trainers in Togo, 11 of whom have completed their qualification by leading a training course with a mentor. However, the current number of trainers is insufficient to expand the program in the countries or across the region; training and equipping more trainers is necessary to expand good-quality counseling and PPIUD services during scale-up.

### Evidence Can Help Advocate Expansion

Dissemination of positive program experiences at conferences has led to expansion of the initiative in 2 of the original 5 countries. Seeing initial program successes in Benin and Niger, each country’s MOH worked with the regional UNFPA mission in collaboration with in-country UNFPA offices to expand the training to an additional 4 sites in each country. This realized the planning team’s vision that countries would respond to positive data, assume ownership for their programs, and seek collaborations to help them showcase their activities and expand their programs. Expanding training to these additional sites in Benin and Niger has produced 20 trained providers on PPFP counseling in each country, and 13 and 15 providers for PPIUD insertion, respectively. Replicating and scaling up this model will require building local capacity for implementation to reduce the cost of the program model over the long term.

### Ensuring Availability of Equipment and Supplies Avoids Service Delivery Delays and Interruptions

Procuring the equipment for PPIUD services can be challenging, and delays cause unnecessary interruptions in service delivery. Sending equipment and supplies home with trainees, including adequate numbers of PPIUD instrument kits (PPIUDs themselves are procured along with other contraceptive methods) and an anatomical model for each facility, allowed teams to begin providing the service immediately. Working with the same tools in their home country with which they had trained also gave them additional confidence.

Because the PPIUD service was new, there was an anticipated lag in caseload until sufficient demand was generated. Having an anatomical model available allowed trainees to maintain their skills and also to demonstrate the procedure to their peers in their facility. Some trainees were so enthusiastic that they trained their colleagues immediately upon their return. This may explain how some sites rapidly ramped up PPIUD services, although informal peer-to-peer training may reduce the quality of skills acquisition. Any quality concerns may be mitigated as the body of regional trainers is expanded to provide further transfer-of-learning visits and oversight to maintain the high quality of services.

**Figure:**
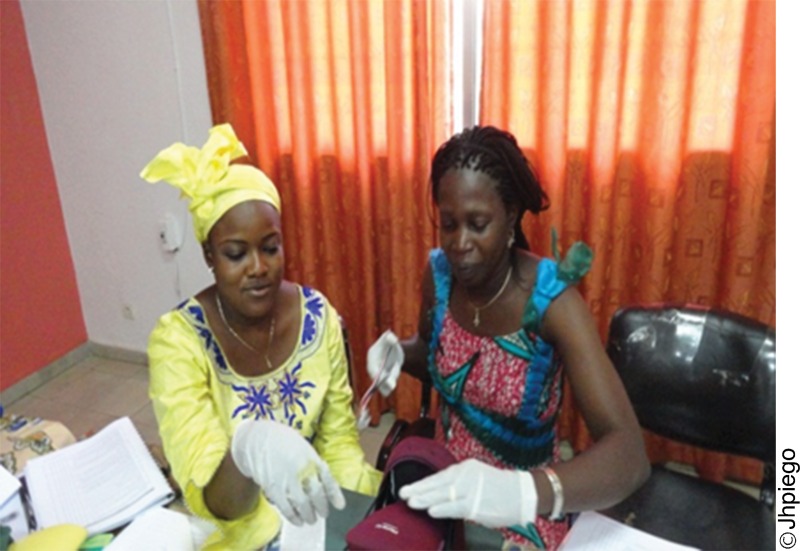
A trainee practices postpartum IUD insertion using an anatomical model.

## ONGOING AND FUTURE CHALLENGES

While these early results are encouraging, uptake has not occurred evenly in the initiative countries, and numbers of insertions per trained provider are not as high as some other PPIUD programs have documented.^21^ The initiative plans to overcome the challenges experienced in the first months of implementation through (1) better communication across the service delivery continuum, (2) advocating the program to the MOH with consistent and accurate data collection and monthly reporting, and (3) creation of a system to avoid stock-outs of essential PPIUD supplies and contraceptives.

### Service Delivery Coordination

Matching demand created for PPFP services with good-quality PPFP service delivery, particularly for PPIUD insertion, requires coordination between antenatal clinics, labor and delivery, family planning units, and postnatal clinic care. Such coordination is typically poor. With PPFP, the optimal time to discuss contraception is during the antenatal period, when a bond can be established between the client and the provider and the client has time to consider her contraceptive options. Not offering PPFP counseling during pregnancy is a missed opportunity, as antenatal counseling gives women time to decide among their family planning options free of the time pressure, the pain of giving birth, and the demands of caring for a new baby. Once admitted to a facility for delivery, women in the early stages of labor may be able to make an informed choice; women in active labor may not.

The ideal time to counsel women about PPFP is during the antenatal period when bonds between the client and provider can be established and the client has time to consider her options.

One way to encourage better coordination across the antenatal–labor-and-delivery–postnatal care continuum would be to note whether a woman has received PPFP counseling and her method choice on the antenatal clinic card. This would potentially improve communication between antenatal and delivery services. Many successful PPFP/PPIUD interventions have used a rubber stamp on the antenatal care card to serve this purpose. Another way to ensure a continuum of care is to extend communication beyond the delivery room to the family planning clinic: women who receive PPIUDs need to know where to go for family planning follow-up visits, and family planning clinics need a record of which contraceptive method women are using.

### Collection and Dissemination of Data

Other PPIUD programs in low-income countries have confirmed that data can be a powerful tool for advocacy,^1^ and maximizing collection and dissemination of data is important to make the case for scale-up of these programs, as seen in the examples of Benin and Niger. The global climate has shifted in favor of reinvigorating family planning, there is strong global agreement that PPFP and PPIUD services are critical strategies to reduce unmet need, and there are capable institutions equipping experts to provide the service regionally—all favorable conditions to convince national governments to take ownership for PPFP/PPIUD programs.^22^ Generating political momentum within the MOH requires credible indicators for PPFP and PPIUD services that demonstrate the safety, acceptability, and cost-effectiveness of PPIUD services.

Generating political momentum within MOHs for PPFP and the PPIUD requires credible data demonstrating safety, acceptability, and cost-effectiveness of the approach.

The continued success of this initiative relies on careful recordkeeping, regular monitoring and feedback to improve quality, and strategic use of data to make the case for scale-up in the countries and throughout the region. Service delivery statistics that demonstrate the safety and acceptability of PPIUDs can correct misconceptions of decision makers—who may associate IUDs with high expulsion rates or other adverse consequences—and build support for PPIUD interventions. However, due to resource constraints, data collection and management tools—such as logbooks to record counseling, insertions, complications, and follow-up visits—are often poorly used. Deficiencies in data management also may contribute to loss to follow-up if facilities fail to record clients’ contact information or if they lose the contact information.

The initiative has encouraged all participating sites to report monthly PPIUD service delivery data from their standardized logbooks to their MOH, even though the indicators are not yet included in any of the countries’ HMIS. Data collection tools using new technology, such as smartphones or tablets, can also help identify and resolve issues, such as PPIUD complications, early. We plan to conduct research to determine how the HMIS can and should track PPFP/PPIUD uptake, and we plan to advocate the addition of PPFP/PPIUD indicators to the HMIS.^1^

### Systems to Avoid Stock-Outs

Failing to have a range of LARCs available in sufficient quantities at every health facility offering PPFP/PPIUD is a missed opportunity. Particularly where demand is being generated during antenatal and postnatal visits, facilities should anticipate demand outpacing available equipment and commodities.

Labor and delivery units have traditionally not offered family planning, but they could order contraceptives through the family planning unit when needed. Opening this procurement channel requires improved communication between labor and delivery units and family planning units. Alternatively, labor and delivery units could develop their own procurement mechanism and system to stock contraceptives and avoid stock-outs. As the initiative matures, we are working to develop effective systems to prevent stock-outs of supplies and commodities in the delivery room and during postpartum services.

### Research to Understand Barriers to PPFP/PPIUD Uptake

Uptake of the PPIUD and service expansion has not occurred evenly across the region. Some countries have been slower to introduce PPIUD services while others, such as Chad, have not yet begun offering services despite participating in provider training. We encountered no provider bias against IUD insertion, although it has been documented in other settings.^23^ Rather, any initial reluctance in the countries where the initiative is actively providing services appeared to be due to an inability to perform the procedure and was overcome through training. Low ANC coverage (39%) may also partially explain the lack of success in Chad.^17^ While the cost of contraceptives can be a barrier, contraceptives are heavily subsidized in all countries participating in this initiative, so cost is unlikely to impede uptake.

Cultural and religious objections to family planning and lingering misconceptions about PPIUDs may contribute to low uptake.^7^^,^^24^ A study in Tanzania found that partner disapproval contributed to women’s lack of follow-through on PPFP after delivery.^25^ In Niger, we documented two cases of removal based on partner disapproval, which indicates that this may be an important factor limiting uptake in some regions. Evidence from Nigeria suggests that multiple antenatal counseling sessions lead to greater use of PPFP.^26^ Understanding the reasons for differences in uptake of the PPIUD and programmatic best practices to overcome cultural and religious barriers are areas for research that may become more relevant as the initiative expands.

## STRENGTHS AND LIMITATIONS

Few reports document the inter-organizational effort to introduce and scale up PPFP counseling and PPIUD services. Our account provides a needed example for future similar programs in other regions. As discussed above, these early findings are too limited to draw confident conclusions about the impact of the program on contraceptive prevalence rate or quality of care (e.g., the expulsion rate data in the Table are a weak indicator of quality of care, and we have no measures of quality of counseling). Additionally, we cannot determine the reasons for the uneven uptake of services across various countries: these may represent cultural barriers, failures in demand generation, or differences in provider motivation and performance, among other reasons. Collecting more and better data on service delivery and quality will be a primary focus of the initiative moving forward. The Box details these and other challenges that the initiative hopes to address.

BOX. **Priority Research Areas of Factors Affecting Use of Postpartum IUD Services**

- Identification of effective recordkeeping and communication technologies to improve continuum of care for PPFP/PPIUD services from antenatal care through delivery, postpartum visits, and family planning clinic visits
- Determination of ways to routinely collect data on service delivery and quality, including total number of women counseled on PPFP and the PPIUD, timing of the counseling, and user satisfaction
- Inclusion of indicators to track PPFP/PPIUD uptake in the HMIS
- Implementation of optimal procurement systems to avoid stock-outs in labor and delivery units, coordinated with demand promotion
- Identification of cultural and religious reasons for differences in uptake of PPIUD services, and best practices to address any misconceptions about the PPIUD

## CONCLUSION

Our initiative’s early successes introducing PPFP and PPIUD services in West and Central Africa illustrate how the seed of a timely idea can be tended with supportive and strategic collaboration between donors, country governments, and a regional body of expert trainers. This collaboration generated momentum to implement a regional model of high-quality PPFP/PPIUD service provision. Ensuring that practitioners are competent and equipped with all the supplies they need leads to better service delivery outcomes, which can ignite political will to expand PPFP/PPIUD services. Evidence from Benin and Niger demonstrate that positive service delivery outcomes can be used to advocate program expansion and scale-up. To achieve the goals of this initiative, we are working to ensure proper data collection, regular reporting of these data to engage MOH and funding agencies, and creation of a system to prevent stock-outs of PPIUD instruments and contraceptives. Our experience aligns with lessons from other PPFP and PPIUD programs: rigorous training incorporating current clinical and program learning, good recordkeeping in facilities, and sustained regional supportive supervision are essential.

To achieve the PPFP/PPIUD initiative goals, we are working to ensure proper data collection, regular data reporting to engage MOH and funding agencies, and prevention of PPIUD instrument and contraceptive stock-outs.

Although the seed has been planted, much work remains for availability of PPFP and PPIUD services to blossom across the region. PPFP and PPIUD indicators need to be incorporated into every country’s HMIS, and facilities where these services are provided need to be supported to maintain service and data management quality as the ranks of trained providers grow. Connecting antenatal visits with labor and delivery and family planning units through counseling and provision of family planning is essential to ensure continuity across the continuum of reproductive health services.

Despite the challenges, this model has the potential to lead to a paradigm shift for family planning services in West and Central Africa. Fruitful South–South collaborations, improved data collection and reporting in facilities, and sharing program experiences with national MOHs and stakeholders across the region make the case for initiatives like this one to become embedded in countries’ long-term family planning program frameworks.

## Notes

**Peer Reviewed**
